# Exosomal miR-9 inhibits angiogenesis by targeting MDK and regulating PDK/AKT pathway in nasopharyngeal carcinoma

**DOI:** 10.1186/s13046-018-0814-3

**Published:** 2018-07-13

**Authors:** Juan Lu, Qi-Hui Liu, Fan Wang, Jia-Jie Tan, Yue-Qin Deng, Xiao-Hong Peng, Xiong Liu, Bao Zhang, Xia Xu, Xiang-Ping Li

**Affiliations:** 1 Department of Otolaryngology-Head and Neck Surgery, Nanfang Hospital, Southern Medical University, Guangzhou, China; 2 School of Public Health and Tropical Medicine, Southern Medical University, Guangzhou, China; 3 Department of Otolaryngology-Head and Neck Surgery, Guangzhou General Hospital of People’s Liberation Army of China, Guangzhou, China

**Keywords:** Exosome, miR-9, Angiogenesis, MDK, Nasopharyngeal carcinoma

## Abstract

**Background:**

Exosomes are small vesicles containing a wide range of functional proteins, mRNA and miRNA. Exosomal miRNAs from cancer cells play crucial roles in mediating cell-cell communication and tumor-microenvironment cross talk, specifically in enabling metastasis and promoting angiogenesis. We focused on miR-9 that was identified as a tumor suppressor previously in nasopharyngeal carcinoma (NPC) tumorigenesis.

**Methods:**

Differential centrifugation, transmission electron microscopy and nanoparticle tracking analysis were used to isolate and identify exosomes. Quantitative PCR and western blotting analysis were used to detect miR-9, pri-miR-9, CD63, TSG101, MDK, P70S6K P-Ser424 and PDK1 P-Ser241 expression. Laser confocal microscopy was used to trace exosomal miR-9 secreted by NPC cells into HUVECs. The effect of exosomal miR-9 on cell migration and tube formation of HUVECs in vivo and vitro was assessed by using migration assay, tube formation assay and matrigel plug assay, respectively. Bioinformatics analysis and luciferase reporter assay were utilized to confirm the binding of exosomal miR-9 to the 3′untranslated region (3′-UTR) of MDK, while Phosphorylation Array was performed to identify AKT Pathway in HUVECs treated with exosomal miR-9. Furthermore, Immunohistochemistry (IHC) and in situ hybridization (ISH) was used to detected miR-9, CD31 and MDK expression in human NPC tumor samples.

**Results:**

NPC cells transfected with miR-9-overexpressing lentivirus, released miR-9 in exosomes. Exosomal miR-9 directly suppressed its target gene - MDK in endothelial cells. Mechanistic analyses revealed that exosomal miR-9 from NPC cells inhibited endothelial tube formation and migration by targeting MDK and regulating PDK/AKT signaling pathway. Additionally, the level of MDK was upregulated in NPC tumor samples and was positively correlated with microvessel density. Notably, the level of exosomal miR-9 was positively correlated with overall survival, and MDK overexpression was positively associated with poor prognosis in NPC patients, suggesting the clinical relevance and prognostic value of exosomal miR-9 and MDK.

**Conclusions:**

Taken together, our data identify an extracellular anti-angiogenic role for tumor-derived, exosome-associated miR-9 in NPC tumorigenesis and prompt further investigation into exosome-based therapies for cancer treatment.

**Electronic supplementary material:**

The online version of this article (10.1186/s13046-018-0814-3) contains supplementary material, which is available to authorized users.

## Background

Nasopharyngeal carcinoma (NPC) is one of common malignant tumors in Southeast Asia, especially in Southern China, with a high rate of local invasion and locoregional lymphatic metastasis [1]. Cervical nodal metastasis is a frequent clinical feature in NPC, occurring in roughly three-quarters of all patients [2]. Because of the improvement of radiotherapy and chemotherapy, excellent local control has generally been achieved, but distant failure is a key challenge, and the treatment of metastatic NPC remains disappointing. Therefore, better understanding of the pathogenesis and metastatic mechanisms is essential for the development of novel therapeutics for NPC.

Exosomes are small vesicles ranging in size between 30 and 150 nm, which have recently been recognized as critical players of intercellular communication [3]. Recent studies have revealed that exosomes play crucial roles in mediating tumor-microenvironment cross talk through the horizontal transfer of proteins, mRNAs and microRNAs (miRNAs) [4]. Tumor-derived exosomes contribute to the development of immunosuppression and promote tumor growth and metastasis. By stimulating neoangiogenesis, activating mesenchymal stroma cells, and remodeling extracellular matrix, they contribute to the formation of a pre-metastatic niche and establish suitable microenvironments in distant metastatic sites [5, 6]. Therefore, these vesicles represent a rich source of novel biomarkers for cancer diagnosis and prognosis.

Pathological angiogenesis is a hallmark of cancer. In addition to the role of exosomes in promoting tumor metastasis, new evidence supports the idea that exosomal miRNAs stimulate angiogenesis by facilitating neoplasia-to-endothelial cell communication. It has been shown that miRNA-enclosed exosomes derived from cancer cells could interact with endothelial cells and thereby stimulate endothelial cell proliferation, migration and tube formation [7]. Umezu et al. found that the exosome, derived from K562 cells with enforced miR-92a expression, did not affect the growth of human umbilical vein endothelial cell line (HUVECs) but did enhance endothelial cell migration and tube formation [8]. Zhang et al. also showed that THP-1 cells selectively packaged miR-150 into microvesicles and actively secreted them into the extracellular environment, and extracellular miR-150 could then enter endothelial cells and enhance cell migration [9]. However, no functional evidence of exosomal miRNAs has been documented in NPC angiogenesis.

Our previous studies found that ectopic expression of miR-9 dramatically inhibited tumor growth and metastasis in NPC [10], and plasma miR-9 might serve as a useful biomarker to predict NPC metastasis and to monitor tumor dynamics [11]. However, another study found that tumor-secreted miR-9 promoted endothelial cell migration and angiogenesis by activating the JAK-STAT pathway in colorectal cancer and melanoma [12]. These findings suggested that the contribution of miR-9 to angiogenesis seemed controversial. Here, we investigated the role of exosomal miR-9 in NPC angiogenesis.

## Methods

### Clinical samples and cell culture

The NPC biopsy specimens and serum samples (*n* = 110) were collected from Nanfang Hospital between 2007 and 2015. All samples were pathologically reassessed. The clinical characteristics of the study participants were presented in Additional file 1: Table S1. None of the patients had received radiotherapy or chemotherapy before biopsy and blood sampling. The clinical classification for NPC was based on the seventh edition UICC/AJCC staging system. All patients were treated with a uniform protocol of image-guided intensity modulated radiotherapy or/and cisplatin-based concurrent chemotherapy following induction chemotherapy according to National Comprehensive Cancer Network (NCCN) Guidelines. Healthy donors were recruited, and normal biopsies of the nasopharynx were obtained from every donor and used as healthy control. Informed consent was obtained from all individuals, and the research protocols were approved by the Ethics Committee of Nanfang Hospital. The approval number is NCT01171235. The median follow-up was 60 months. The 5-year overall survival rate was 71.8%.

Human NPC cell lines (5-8F and CNE1), HUVECs and human embryonic kidney 293 T cells (HEK293T) were purchased from the Cell Bank of the Chinese Academy of Sciences (Shanghai, China). An immortalized nasopharyngeal epithelial cell NP69 was kindly provided by cancer center of Sun Yat-sen University. NPC cell lines were cultured in RPMI1640 supplemented with 10% heat-inactivated exosome depleted fetal bovine serum (Gibco) at 37 °C in a humidified atmosphere containing 5% CO2. HUVEC cells were cultured in endothelial basal medium (EBM-2) supplemented with 10% fetal bovine serum. NP69 was cultured in Keratinocyte-SFM (Invitrogen) supplemented with bovine pituitary extract (BD Biosciences). HEK293T cells were cultured in Dulbecco’s modified Eagle’s medium (DMEM; Invitrogen). The PDK1 inhibitor OSU-03012 was purchased from Selleckchem (Houston, TX).

### Exosomes isolation, transmission electron microscopy and nanoparticle tracking analysis

Exosomes were isolated from the plasma of healthy controls or NPC patients and from cell culture medium by differential centrifugation as previously described [13]. Briefly, collected culture supernatants or plasma samples were differentially centrifuged at 300×g, 1200×g, and 10,000×g for 3 h, and the supernatant was filtered and ultracentrifuged at 110,000×g for 3 h (all steps were performed at 4 °C). Exosomes were collected from the pellet and resuspended in phosphate-buffered saline (PBS).

Exosomes were prepared and fixed as described previously [14]. The samples were observed with a transmission electron microscope (H-7650, HITACHI, Japan) at an acceleration voltage of 80 kV.

Measurements for nanoparticle tracking analysis (NTA) were performed using the Nanosight NS3000 system (Nanosight, Amesbury, United Kingdom) equipped with a blue laser (405 nm). Exosomes were illuminated by the laser, and their movement under Brownian motion was recorded in 9 s sample videos which were analyzed with NTA analytical software (version 2.3, Nanosight). The capture settings and analysis settings were done manually according to the manufacturer’s instructions.

### Cy3-labeled pre-miR miRNA trasnfection, exosomes labeling and confocal microscopy

Pre-miR miRNA precursor (hsa-miR-9, Ambion, Austin, TX) was labeled with Label IT siRNA Tracker Cy3 Kit, according to the manufacturer’s instructions (Mirus, Madison, WI, USA). 5-8F cells were transfected with 100 nM of Cy3-labeled pre-miR miRNA precursor using HiPerFect (Qiagen, Dusseldorf, Germany). After incubation for a day, the culture medium was collected and used for exosome preparation. The exosomes including Cy3-miR-9 were labeled using the green lipophilic fluorescent dye PKH67 (Sigma-Aldrich, St. Louis, MO, USA). The PKH67-labeled exosomes including Cy3-miR-9 were incubated with cultured HUVEC cells. After incubation, HUVEC cells were washed and visualized under confocal microscopy (FV10i, Olympus, Japan). 4′6-diamidino-phenylidole (DAPI, Abbott, USA) was used for nuclear staining.

### Migration assay and tube formation assay

The migration ability of HUVEC cells was tested with BD Transwell invasion assay inserts, according to the manufacturer’s protocol. HUVEC cells were placed in the upper chamber of a Transwell coated with Matrigel. The chamber was placed in a 24-well culture dish. After incubation, the cells that did not invade through the pores were removed by a cotton swab. Cells on the lower surface of the membrane were fixed with 4% paraformaldehyde, stained with 0.5% crystal violet and counted.

The formation of capillary-like structures was analyzed as described previously [14]. HUVEC cells (2 × 10^4^ cells/well) were plated on top of Matrigel (300 μL/well) and treated with exosomes (600 μL of exosome fraction/well) derived from 5-8F/con or 5-8F/miR-9 cells. The total tube area was quantified as mean relative tube length obtained from image analysis of five random microscopic fields using Image J software (http://rsb.info.nih.gov/nih-image).

### Matrigel plug assay

Matrigel plug assays were performed as described elsewhere [15]. Briefly, 4 × 10^6^ HUVEC cells were incubated with exosomes derived from 5-8F/miR-9 cells cells or 5-8F/con cells. The cell suspensions were mixed with 500 μl of Matrigel-reduced growth factor (BD Matrigel 356,230) at a ratio of 1:1. Then the cell suspensions were injected subcutaneously in the dorsal region of nude mice (female, 7–8 weeks old, BALB/c). Plugs were recovered 2 weeks later and then embedded in OCT, cryostat sectioned, and stained by hematoxylin and eosin. The vessel area was quantified as mean relative tube length obtained from image analysis of six random microscopic fields using Image J software. For all the experiments, animal handling and experimental procedures were approved by the Animal Experimental Ethics Committee of Southern Medical University.

### Dual luciferase reporter assay

A 300-bp fragment of MDK 3′ untranslated regions (UTR) including wild type (wt) or mutant (mt) miR-9 binding sites were cloned downstream of the firefly luciferase gene in pGL3 vector (Promega). For reporter assays, HUVEC cells were firstly co-transfected with wt or mt MDK 3′ UTR vector and the control vector pRL-CMV [(cytomegalovirus) coding for Renilla luciferase, Promega) using lipofectamine 2000 reagent (Invitrogen). Then the cells were incubated with exosomes extracted from miR-9-overexpressing NPC cells or control cells. Luciferase activity was measured using the Dual-Luciferase Reporter Assay System (Promega, Madison, WI, USA).

### Western blot

The cells were lysed in lysis buffer (Roche, Penzberg, Germany), and equal amounts of protein were separated by SDS-PAGE. The exosome pellets isolated from the same amount of culture medium (10 mL) were lysed in 200 μL of lysis buffer (Roche), and the same amounts of lysate were loaded in each lane of the gels. Immunoblots were probed with antibodies directed against MDK (rabbit monoclonal anti-Midkine, Abcam, Cambridge, MA, USA), P70S6K P-Ser424 (rabbit polyclonal anti-S6 K1, Abcam), or PDK1 P-Ser241 (rabbit polyclonal anti-PDK1, Abcam). CD63 (rabbit monoclonal anti-CD63, Abcam) and TSG101 (rabbit monoclonal anti-TSG101, Abcam) were used as exosomal markers.

The human and mouse AKT Pathway Phosphorylation Array and MAPK Pathway Phosphorylation Array were performed according to the manufacture’s instructions (RayBiotech, Norcross, GA, USA).

### Immunohistochemistry (IHC) and in situ hybridization (ISH)

For quantification of microvessel density (MVD) in NPC tumor samples, the number of blood vessels staining positive for CD31 (1:100 dilution, Abcam, Cambridge, MA, USA) was recorded in ten random fields at 200 magnification. The expression of MDK in NPC tumor samples was examined with IHC as previously described [16]. The antibody was purchased from Abcam (rabbit monoclonal anti-Midkine, Abcam). In situ detection of miR-9 on formalin-fixed paraffin-embedded samples was essentially performed with a miRCURY LNA™ miR-9 detection probe, using an ISH optimization kit (Exiqon, Vedbaek, Denmark) [10]. A scramble-miR probe (Exiqon) was performed as negative control.

### Plasmids, transfection and lentivirus transduction

The lentiviral vector for delivery of pre-miR-9 was described previously [10]. The vector for cDNA delivery of MDK was purchased from GeneCopoeia. The production, purification, and titration of lentivirus were performed as described by Tiscornia et al. [17]. Transient transfection of miR-9 mimic or inhibitor (Ambion, Thermo Fisher Scientific) was performed using Lipofectamine 2000 reagent (Invitrogen), and transient transfection of miR-9 mimic or control into exosomes was performed using Exo-fect exosome transfection kit (System Biosciences). To inhibit MDK expression, siRNA against MDK (Invitrogen) was used. Transient transfection of siMDK was performed using Lipofectamine 2000.

### RNA isolation, reverse transcription and quantitative real-time PCR

Isolation of cellular and exosomal miRNAs was performed using TRIzol reagent (Invitrogen) and Total Exosome RNA and Protein Isolation Kit (Invitrogen) respectively as described previously [14]. MiR-9 expression in both cells and exosomes was performed using a TaqMan microRNA reverse transcription kit and TaqMan microRNA assay kit (Applied Biosystems, Foster City, CA) according to the manufacturer’s instructions. To measure the mRNA levels of MDK and pri-miR-9, total RNA was reversely transcribed using ImProm-II Reverse Transcription System (Promega). Quantitative real-time PCR (qPCR) was performed using SYBR Green PCR master mix (Applied Biosystems). All samples were normalized to internal controls and fold changes were calculated through relative quantification [18].

### Statistical analysis

All statistical analyses were conducted using SPSS 19.0 software (SPSS, Chicago, IL, USA). Data were presented as Mean ± SD of at least three independent experiments. Two-tailed Student’s t test was used for comparisons of two independent groups. Comparisons of multiple independent groups were analyzed using One-way ANOVA followed by a Student-Newman-Keuls test. Survival curves were plotted by the Kaplan-Meier method and compared by the log-rank test. The relationships between CD31 expression and miR-9 or MDK expression were explored by Spearman’s correlation. A two-sided *P* < 0.05 was considered statistically significant.

## Results

### miR-9 is reduced in exosomes derived from cultured NPC cells and plasma samples

**Fig. 1:**
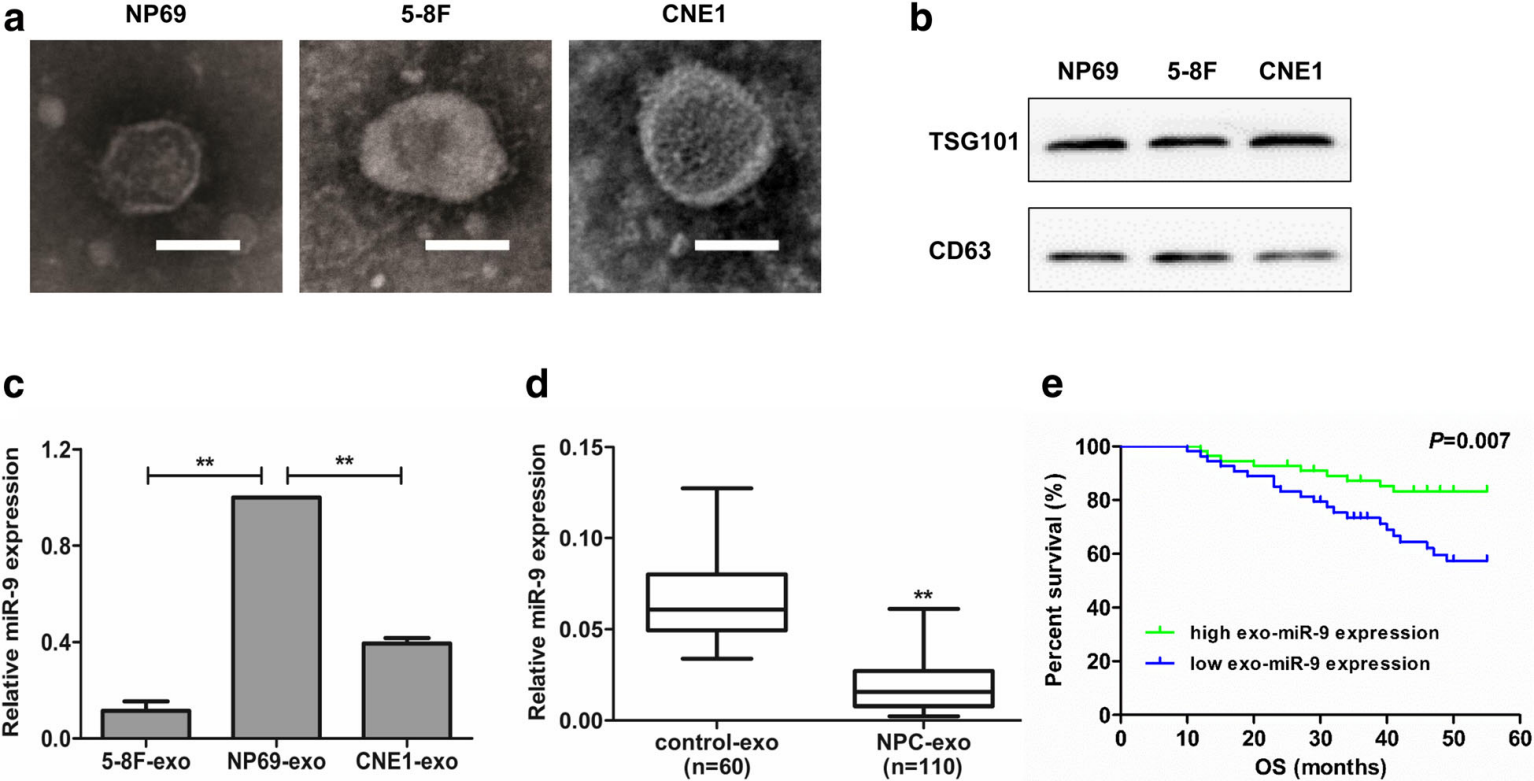
miR-9 was reduced in exosomes derived from cultured NPC cells and plasma samples. **a** Representative images of transmission electron microscopy for exosome derived from NP69, 5-8F and CNE1 cells. Scale bars, 50 nm. **b** CD63 and TSG101 (common exosomal markers) immunoblots of exosomes derived from above three cell lines. **c** The expression of miR-9 in the exosomes derived from NP69, 5-8F and CNE1 cells. **d** Examination of exosomal miR-9 level in plasma samples of NPC patients (*n* = 110) and healthy controls (*n* = 60). **e** A high level of plasma exosomal miR-9 predicted good survival of NPC patients. **, *P* < 0.01

In this study, we chose the NPC cell lines 5-8F and CNE1 as models to investigate tumor-secreted exosomes and miRNAs, and normal NP cell line NP69 was used as a control. Transmission electron microscopy revealed that the size of exosomes was similar between the NPC cell lines and NP69 cells, and each vesicle showed the classic cup-shaped morphology with the common exosomal markers (CD63 and TSG101) (Fig. 1a and b). We then compared the exosomal miR-9 expression with qRT-PCR, and found that miR-9 was reduced in the exosomes derived from NPC cells compared with NP69 cells (Fig. 1c), which was consistent with the data of cellular miR-9 levels in NPC cells [10]. Moreover, we found that exosomal miR-9 levels were significantly lower in plasma samples of NPC patients (*n* = 110) than in those of healthy controls (Fig. 1d). Notably, the data further showed that the plasma exosomal miR-9 level was positively correlated with overall survival in NPC patients (Fig. 1e, *P* = 0.007).

### Exosomal miR-9 is delivered from NPC cells into endothelial cells

**Fig. 2:**
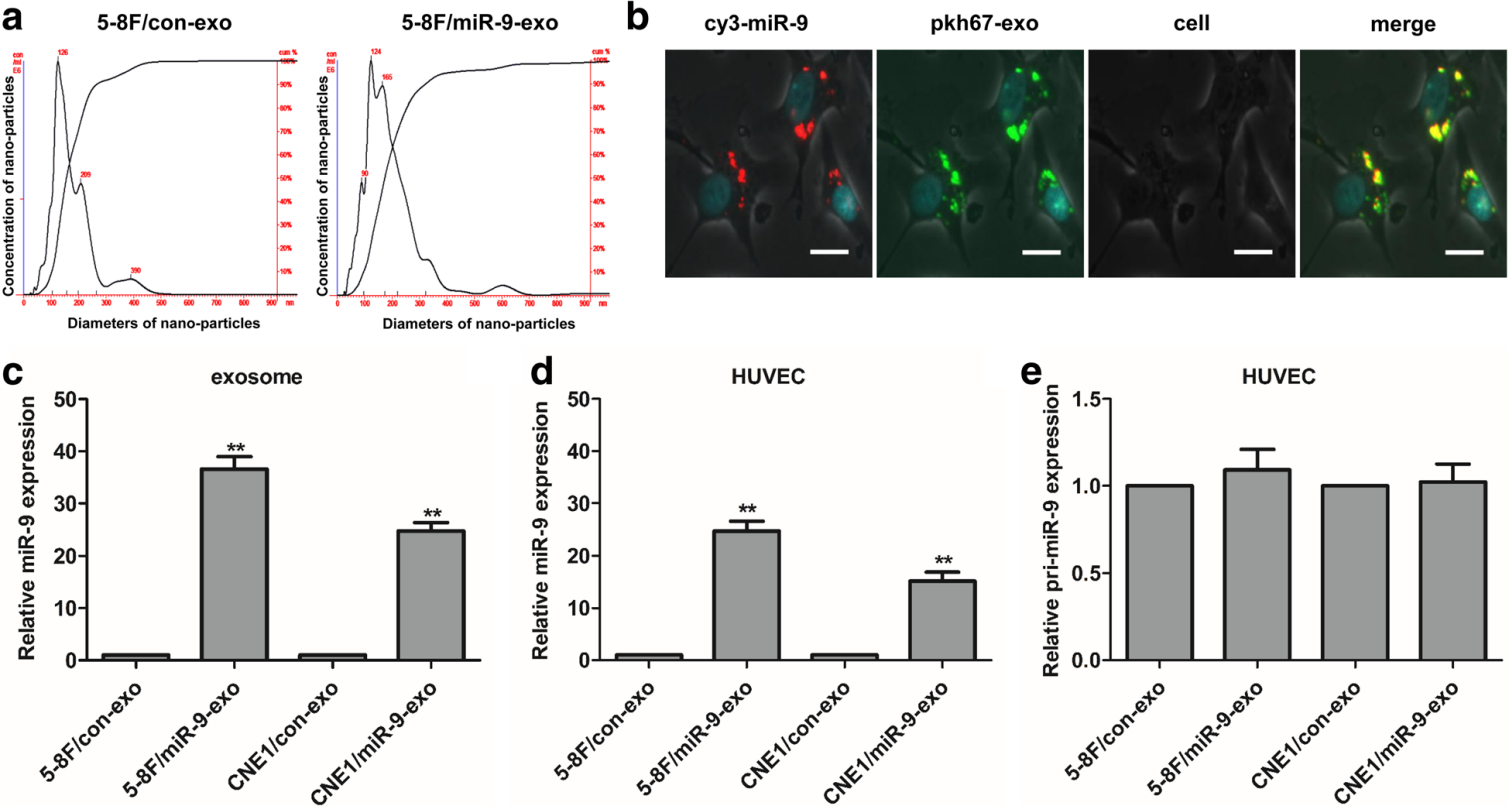
Transfer of miR-9 derived from NPC cells to HUVECs via exosomes. **a** The nanoparticle concentration and size distribution of the exosomes derived from 5-8F/con and 5-8F/miR-9 cells. **b** Transfer of miR-9 derived from NPC cells to HUVECs via exosomes. HUVECs were cultured with PKH67-labeled exosomes derived from the parental cells (5-8F) transfected with Cy3-pre-miR-9. The Cy3-miR-9 signals were detected in the cytoplasm and nucleus of HUVECs (red), and green signals indicated PKH67-labeled exosomes. Cy3-miR-9 signals are colocalized with PKH67 in HUVECs (yellow). Nuclear counterstaining was performed using 4′,6-diamidino-2-phenylindole (DAPI) (blue). The scale bar indicated 10 μm. **c** The expression of miR-9 in the exosomes derived from 5-8F/con, 5-8F/miR-9, CNE1/con and CNE1/miR-9 cells. **d** The expression of miR-9 in HUVEC cells which were co-cultured with exosomes derived from 5-8F/con, 5-8F/miR-9, CNE1/con and CNE1/miR-9 cells respectively. **e** The expression of pri-miR-9 in HUVEC cells which were co-cultured with exosomes derived from 5-8F/con, 5-8F/miR-9, CNE1/con and CNE1/miR-9 cells respectively. **, *P* < 0.01

In order to elucidate whether tumor-secreted miR-9 was delivered to endothelial cells via exosomes, 5-8F and CNE1 cell lines were transfected with LV-miR-9 (Additional file 2: Figure S1), then the conditioned medium was collected and processed for exosomal purification. The nanoparticle concentration and size distribution of the exosomes were examined. The results showed that there was no difference between the parental cells (5-8F) and 5-8F/miR-9 in the nanoparticle size distribution and secreted amount of exosomes (Fig. 2a). Then, we visualized the transport of exosomal miR-9 derived from NPC cells into HUVEC. After incubation with PKH67-labeled exosomes derived from 5-8F/Cy3-miR-9 cells, the Cy3-miR-9 signals and PKH67 signals were colocalized in the cytoplasm of HUVEC (Fig. 2b). In exosomes extracted from 5-8F/miR-9 or CNE1/miR-9 cells, levels of exosomal miR-9 was much higher than in those extracted from control cells (Fig. 2c). Treatment of HUVEC with miR-9-overexpressing exosomes induced an increase of the cellular levels of mature miR-9 compared with levels in cells treated with control exosomes (Fig. 2d). In contrast, the expression levels of pri-miR-9 showed no difference in HUVEC treated with miR-9-overexpressing exosomes or control exosomes (Fig. 2e), indicating that the upregulated expression of miR-9 in HUVEC was induced by exosome-mediated miR-9 transfer and not pri-miR-9 transcription.

### Exogenous miR-9 inhibits recipient HUVEC cell migration and tube formation

**Fig. 3:**
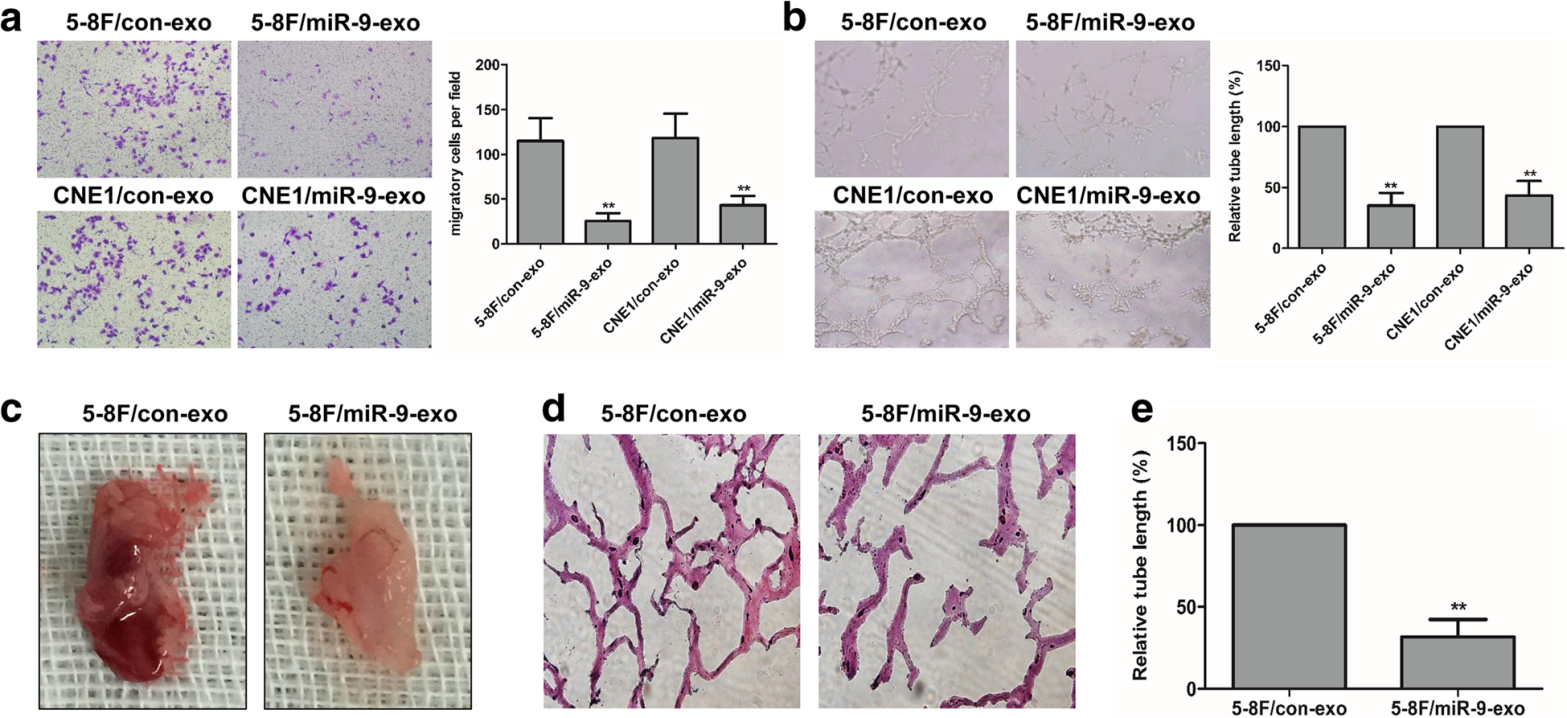
Exogenous miR-9 inhibited recipient HUVEC cell migration and tube formation. Exosomes were extracted from the medium of 5-8F/con or 5-8F/miR-9 cells and co-cultured with HUVECs. Then cell migration was measured by Transwell migration assay as shown in (**a**). Representative pictures of transwell migration (Left) were taken at 16 h postplating and quantified for migratory cells (Right). Tube formation of HUVECs was examined by in vitro tube formation assay as shown in (**b**). Representative pictures of tube formation (Left) were taken at 18 h postplating and quantified for tubule length (Right). Tube formation of HUVECs was also examined by in vivo matrigel plug assay. Representative pictures of matrigel plug were taken at 2 weeks postplating as shown in (**c**). Representative pictures of tube formation were shown in (**d**) and quantified for tubule length as shown in (**e**). **, *P* < 0.01

Endothelial cell migration and tube formation were important steps of angiogenesis. Thus, to confirm the role of exosomal miR-9 in angiogenesis, we studied the effect of exosomal miR-9 on the migration and tube formation of HUVEC. HUVEC cells were added with the respective exosomes, with or without miR-9 overexpression, into the upper chamber of the transwell and incubated for 24 h. After incubation, the number of cells that migrated to the other side of the membrane was counted. Treatment with miR-9-overexpressing exosomes led to a significant decrease in migration of HUVEC, compared to exosomes derived from negative control NPC cells (Fig. 3a). The in vitro endothelial tube formation assay showed that exosomes from miR-9-overexpressing NPC cells significantly inhibited tube formation of HUVEC compared with the control (Fig. 3b). To further confirm the anti-angiogenic effect of exosomal miR-9, miR-9 mimic or control was directly transfected into exosomes derived from 5-8F cells. Then, HUVEC cells were added with the respective exosomes, with or without miR-9 overexpression. Following miR-9-overexpressing exosomes treatment, transwell migration assay and tube formation assay were performed. The data demonstrated that miR-9-overexpressing exosome treatment significantly inhibited cell migration and tube formation of HUVEC compared with control (Additional file 3: Figure S2). Taken together, these results clearly demonstrated that secreted miR-9 present in exosomes of NPC cells could be effectively delivered to cultured cells, where it functioned as an endogenously anti-angiogenic miRNA.

To further confirm the anti-angiogenic role of exosomal miR-9, we performed an in vivo matrigel plug assay to detect the newly-formed blood vessels in the transplanted gel plugs in nude mice (Fig. 3c). The relative tube length of the neovessels line in Matrigel plugs was quantified (Fig. 3d). The data revealed that the plugs containing the exosomes derived from 5-8F/miR-9 significantly reduced the relative tube length compared with control (Fig. 3e). These additional experiments provided evidence of important roles for exosomal miR-9 derived from NPC cells on angiogenesis in vivo.

### Exosomal miR-9 derived from NPC cells targets MDK and regulates PDK/AKT signaling pathway

**Fig. 4:**
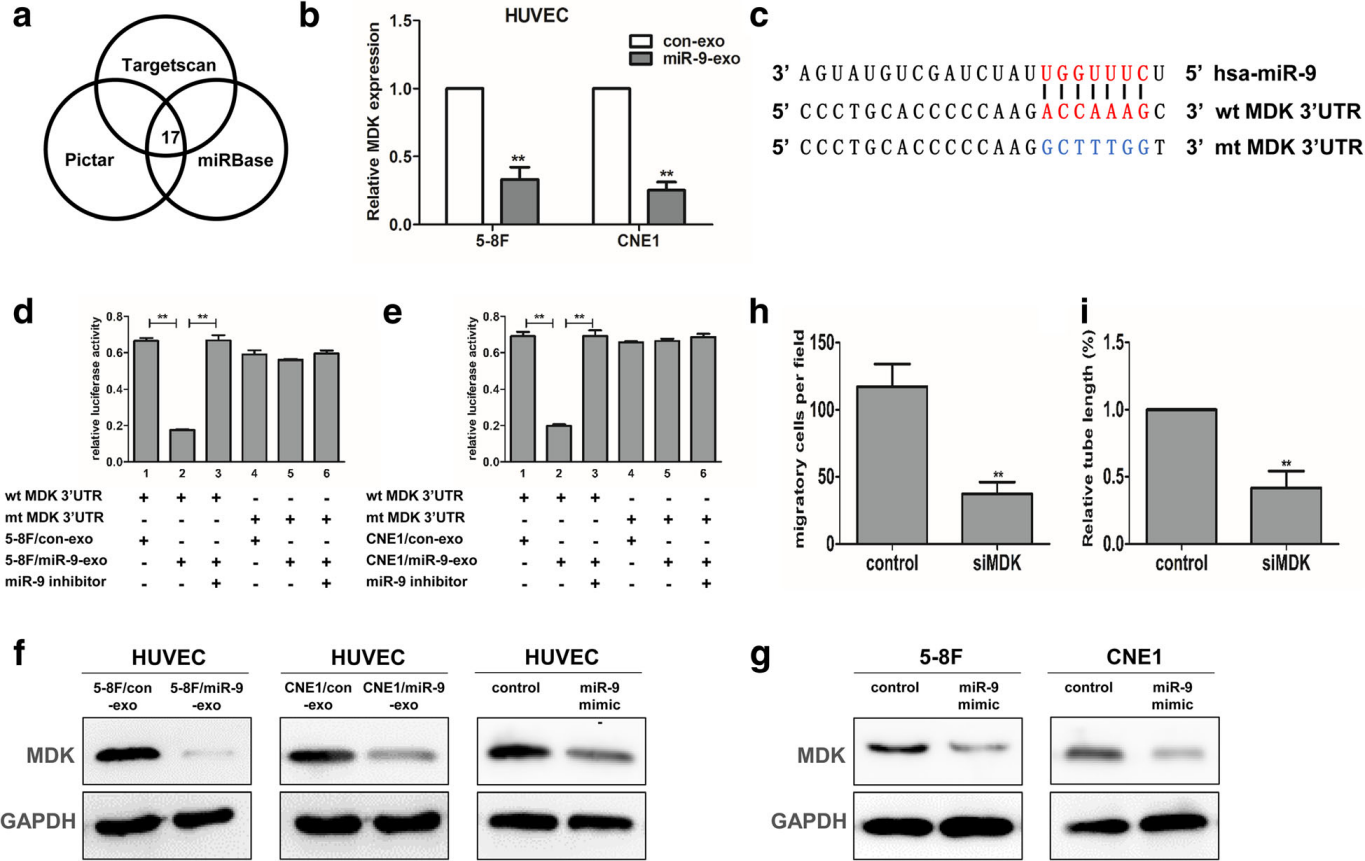
Exosomal miR-9 derived from NPC cells targeted MDK in endothelial cells. **a** Seventeen angiogenesis-related factors were overlapped predicted by Targetscan, miRBase and Pictar. **b** The mRNA level of MDK in HUVEC cells after miR-9-overexpressing exosomes treatment. **c** Diagram of MDK 3′UTR-containing reporter constructs. **d** Luciferase reporter assays in HUVECs, with cotransfection of wt or mt MDK 3′UTR and exosomes derived from 5-8F/con or 5-8F/miR-9 cells as indicated. These experiments were performed in triplicate, and the results were shown as Mean ± SD. **e** Luciferase reporter assays in HUVECs, with cotransfection of wt or mt MDK 3′UTR and exosomes derived from CNE1/con or CNE1/miR-9 cells as indicated. **f** The protein levels of MDK in HUVEC cells after treatment with different exosomes as indicated. The intensity of each band was normalized by GAPDH. **g** The protein levels of MDK in NPC cells after transfection with miR-9 mimic or negative control. **h** After direct knockdown of MDK in HUVEC cells by siRNA, cell migration was measured and quantified by Transwell migration assay. **i** After direct knockdown of MDK in HUVEC cells by siRNA, tube formation of HUVECs was examined by in vitro tube formation assay and quantified for tubule length. **, *P* < 0.01

To clarify the molecular mechanisms by which exosomal miR-9 inhibited angiogenesis in endothelial cells, we performed in silico analysis to predict possible miR-9 targets using database resources including Targetscan, PicTar and miRanda. Each program yielded a large number of targets. However, the 17 top candidates were common to all these three methods (Fig. 4a). Interestingly, MDK was identified as one of the top candidates, which had been shown to enhance the angiogenic and proliferative activities of cancer cells. We examined the mRNA level of MDK in HUVEC cells following miR-9-overexpressing exosomes treatment. The results showed that miR-9-overexpressing exosomes treatment indeed reduced the mRNA level of MDK in HUVEC cells (Fig. 4b). Therefore, we subsequently investigated whether MDK was a direct target of miR-9. The target sequence of MDK (Fig. 4c) 3′UTR (wt 3′UTR) or the mutant sequence (mt 3′UTR) was cloned into a luciferase reporter vector respectively, HUVEC cells were then transfected with wt or mt 3′UTR vector and co-cultured with the respective exosomes with or without miR-9 overexpression. The results showed a more than 1.5-fold decrease of luciferase activity when compared with control (Fig. 4d and e, lanes 1 and 2, *P* < 0.01). The activity of mt 3′UTR vector was unaffected when co-cultured with 5-8F/miR-9-overexpressing exosomes (Fig. 4d and e, lanes 4 and 5). Moreover, transfection of miR-9 inhibitor led to a 3-fold increase of luciferase activity in HUVEC cotransfection with wt 3′UTR vector and miR-9-overexpressing exosomes (Fig. 4d and e, lanes 2 and 3). We also found that MDK protein was reduced when HUVEC cells were co-cultured with miR-9-overexpressing exosomes (Fig. 4f), similar to the results of miR-9 mimic direct transfection into HUVEC cells (data not shown).

To examine MDK expression in NPC cells, we transfected miR-9 mimic or negative control into 5-8F and CNE1 cells. The results showed that transfection of miR-9 mimic could reduce the expression of MDK in NPC cells (Fig. 4g). In contrast, MDK protein was not detected in exosomes derived from 5-8F and CNE1 cells (Additional file 4: Figure S3). The reduction of MDK expression in NPC cells might be induced by endogenous miR-9, and MDK protein of NPC cells did not transfer to the other cells via exosomes. These data indicated that exosomal (exogenous) miR-9 interacted with the MDK 3′UTR to exert translational repression in HUVEC cells. Moreover, direct knockdown of MDK in HUVEC by siRNA (efficient interference of MDK expression was shown in Additional file 5: Figure S4) significantly inhibited cell migration (Fig. 4h) and tube formation (Fig. 4i). To elucidate whether the anti-angiogenic effect of exosomal miR-9 was mediated by repression of MDK in HUVEC cells, we performed a MDK rescue assay. HUVEC cells were infected with LV-MDK for 72 h and followed by treatement with miR-9-ovexpressing exosome. We showed that ectopic expression of miR-9 significantly reversed MDK-induced promotion of cell migration and tube formation (Additional file 6: Figure S5).

**Fig. 5:**
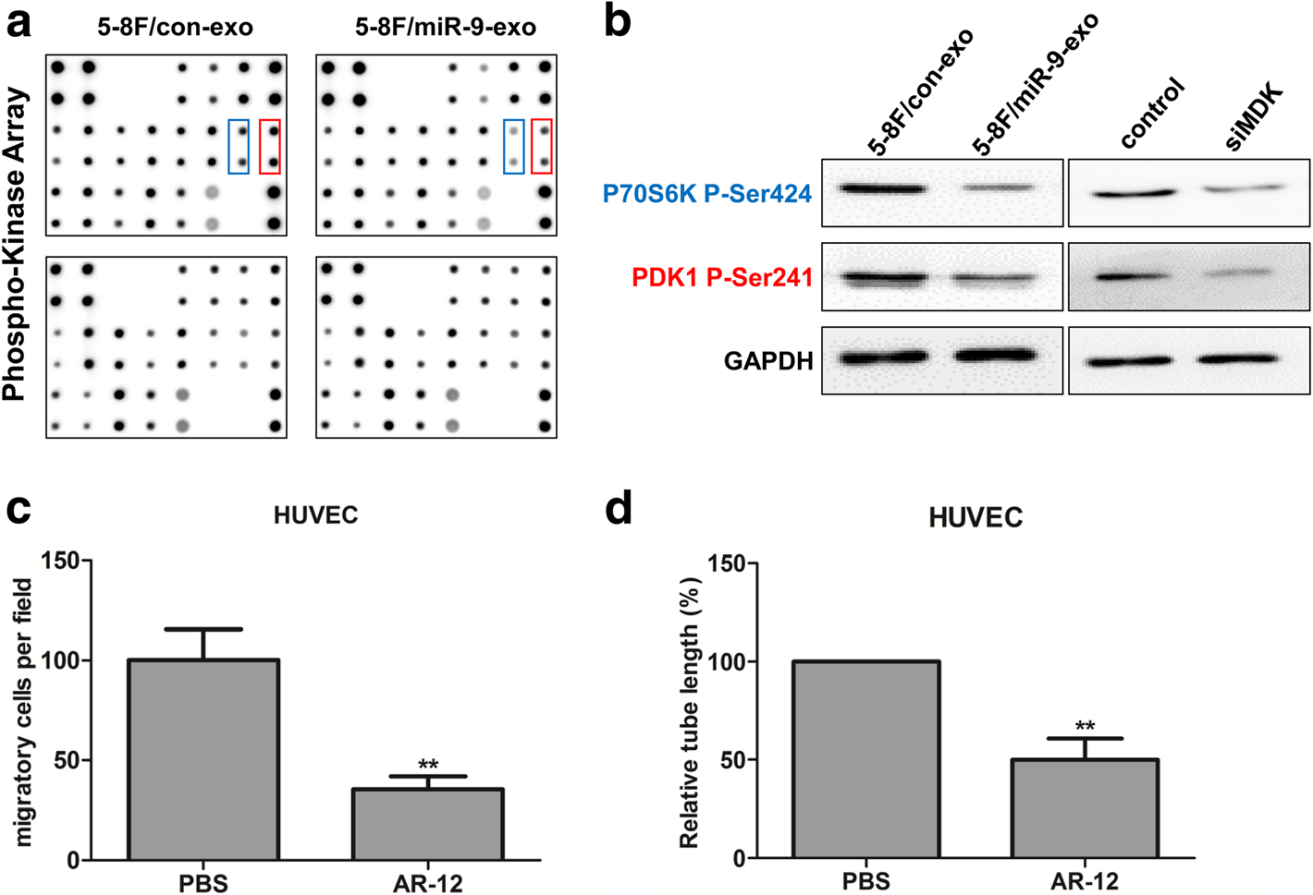
Exosomal miR-9 derived from NPC cells inhibited angiogenesis by regulating the MDK-PDK/AKT signaling pathway. **a** Antibody array analysis of HUVECs co-cultured with exosomes derived from 5-8F/miR-9 or 5-8F/con cells. Blue and red squares indicated more than two-folds of changes in protein phosphorylation. **b** HUVECs were co-cultured with exosomes derived from 5-8F/miR-9 and 5-8F/con cells, or were transfected with siMDK or control, respectively. Then, the expression of P70S6K and PDK1 were analysed by western blot. **c** After inhibiting PDK1 in HUVECs with OSU-03012 (AR-12), cell migration was measured and quantified by Transwell migration assay. **d** After inhibiting PDK1 in HUVECs with OSU-03012 (AR-12), tubule formation of HUVECs was examined by in vitro tube formation assay and quantified for tubule length. **, *P* < 0.01

To investigate the molecular mechanisms by which exosomal miR-9 repressed endothelial cell migration and tube formation, we examined the major signaling pathways involved in endothelial cell function in HUVEC treated with miR-9-overexpressing exosomes. Among 35 components tested, PDK1 and P70S6k exhibited the most marked decrease in phosphorylation (Fig. 5a). Using a different antibody, we confirmed that phospho-PDK1 and phospho-P70S6k was downregulated following miR-9-overexpressing exosomes treatment (Fig. 5b). In agreement with these findings, silencing of MDK expression also inhibited PDK1 and P70S6k phosphorylation in HUVEC (Fig. 5b). We next determined whether interfering pharmacologically with PDK1/AKT pathway could mimic the anti-angiogenic role of miR-9. We used OSU-03012 (AR-12), a potent PDK1 inhibitor. The data showed that OSU-03012 (AR-12) treatment inhibited endothelial cell migration and tube formation (Fig. 5c and d), similar to the role of miR-9-overexpressing exsomes in HUVEC. To further support the link between MDK and PDK/AKT pathway, we performed functional studies to determine whether interfering pharmacologically with PDK1/AKT pathway could rescue the effect of MDK. HUVEC cells were infected with LV-MDK for 72 h and followed by treatement with AR-12. We found that AR-12 treatment significantly reversed MDK-induced promotion of cell migration and tube formation (Additional file 7: Figure S6). Taken together, these data indicated that the inhibitory effect of exosomal miR-9 on endothelial cell was mediated by targeting MDK and regulating PDK1/AKT pathway.

### Expression of miR-9 is negatively correlated with microvessel density in NPC

**Fig. 6:**
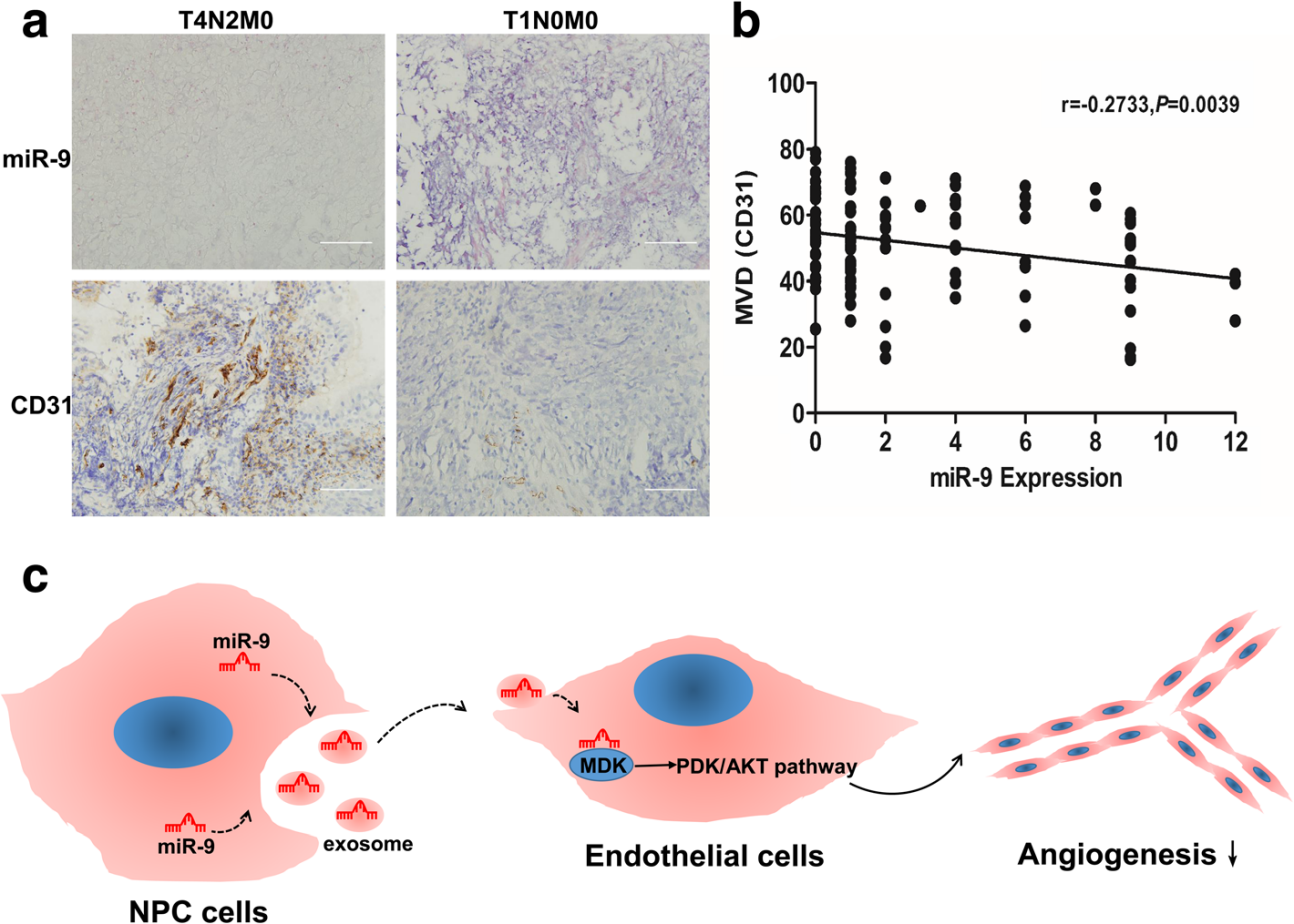
Expression of miR-9 was negatively correlated with microvessel density in NPC. **a** Representative images of immunohistochemical staining for CD31 with low or high levels of miR-9. Scale bars, 100 μm. **b** A negative correlation between miR-9 expression and MVD in NPC tissues. **c** A schematic diagram illustrated how exosomal miR-9 from NPC cells inhibited angiogenesis by targeting MDK-PDK/AKT pathway in NPC. **, *P* < 0.01

We further evaluated the potential associations between miR-9 expression and microvessel density (MVD) in NPC specimens. Tumors with decreased miR-9 expression had significantly greater MVD (Fig. 6a). When the relative expression of miR-9 was plotted against that of CD31 in each NPC sample, a significant negative correlation was found (*r* = − 0.2733, *P* = 0.0039, Fig. 6b). These data provided evidence that miR-9 expression was negatively correlated with tumor angiogenesis in NPC. A summary diagram that outlined this regulatory network was shown in Fig. 6c.

### MDK is overexpressed and positively correlated with microvessel density in NPC

**Fig. 7:**
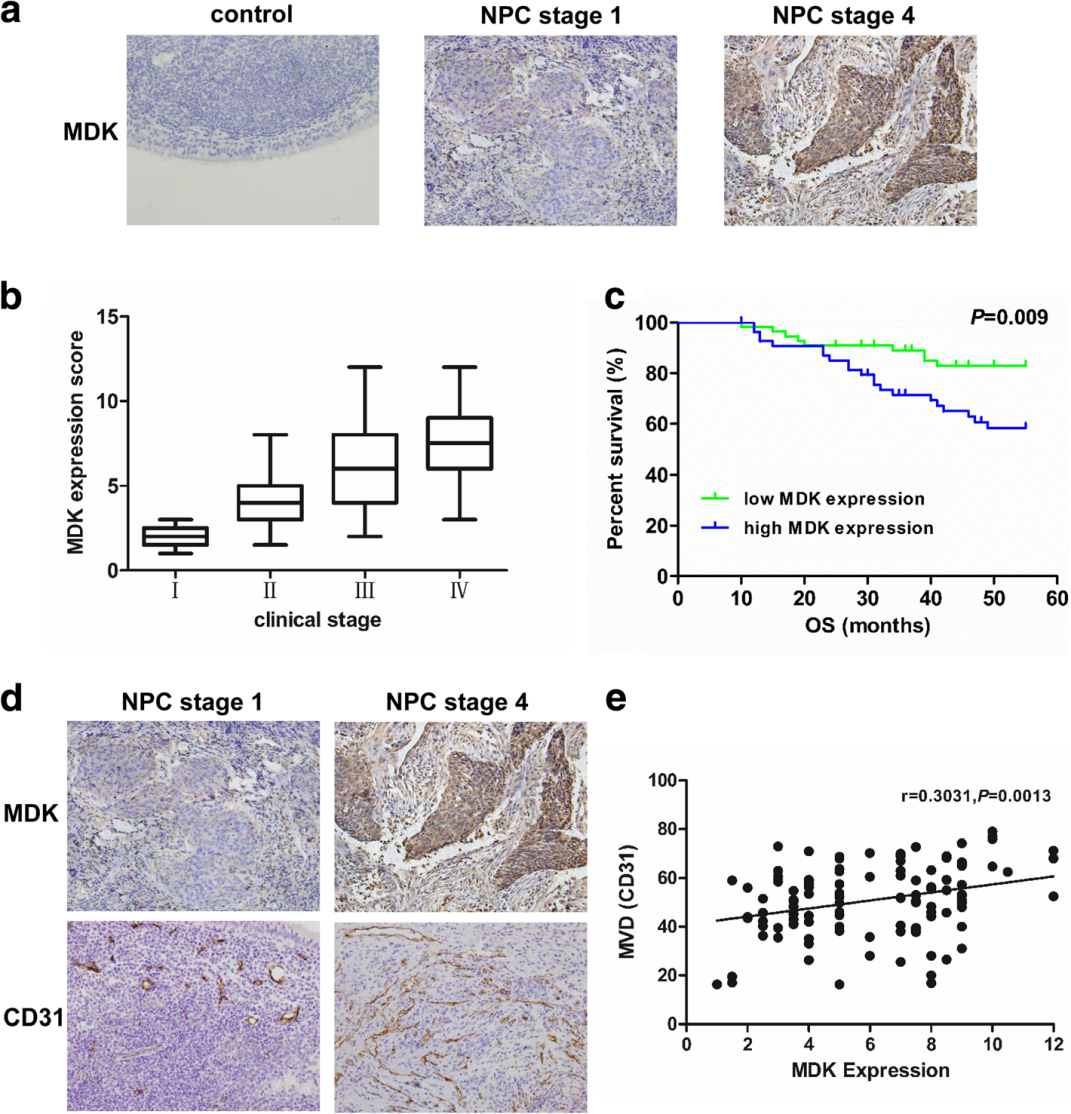
MDK was overexpressed and positively correlated with MVD in NPC. **a** Representative images of immunohistochemical staining for MDK in NPC specimens and healthy control. Magnification, × 20. **b** Examination of MDK expression in the NPC tissues with different clinical stage. **c** A high expression of MDK was significantly associated with a poor OS. **d** Representative images of immunohistochemical staining for MVD with low or high levels of MDK in stage 1 and stage 4 NPC tissues. **e** A positive correlation between MDK expression and MVD in NPC tissues. **, *P* < 0.01

To further investigate the role of MDK in angiogenesis, we first examined the expression of MDK in NPC tissues. The results showed that MDK was highly expressed in NPC samples compared with normal controls (Fig. 7a). Correlation analysis demonstrated that high expression of MDK was positively associated with a more advanced clinical stage (*P* < 0.01, Fig. 7b). Survival analysis showed that high MDK expression was positively associated with poor prognosis (*P* = 0.009, Fig. 7c). We next evaluated potential associations between MDK expression and MVD. Tumors with upregulated MDK expression had significantly greater MVD (Fig. 7d). When the relative expression level of MDK was plotted against that of CD31 in each NPC samples, a significant positive correlation was found (*r* = 0.3031, *P* = 0.0013, Fig. 7e). All these data provided strong evidence that high expression of MDK was closely associated with tumor angiogenesis in NPC.

## Discussion

This is the first study to elucidate NPC cells to endothelial cells communication via an exosomal miRNA, which might be associated with the angiogenic activity of endothelial cells. Firstly, we visualized the transfer of NPC cell-derived miR-9 to HUVECs. Secondly, we found that exogenous miR-9 via exosomal transport could function as an endogenous miRNA in endothelial cells. Finally, we proved that cell-to-cell communication via exosomal miR-9 affected endothelial migration and tube formation through targeting MDK and regulating PDK/AKT signaling pathway. These data indicate that miRNAs originating from cancer cells can regulate the angiogenic activity of endothelial cells.

Mounting evidence suggests the key role of tumor-derived exosomes in modulating angiogenesis in many cancers, such as melanoma [19, 20], leukemia [21], breast cancer [22, 23]. Tumor-derived exosomes release a specific content of miRNAs, mRNAs, and proteins in endothelial cells, therefore modifying angiogenesis. Several reports find that miR-9 functions as a regulator of angiogenesis, and this miRNA has a dual function of pro- or anti-angiogenetic activity by the cooperation of individual target genes in endothelial cells. One study showed that microvesicles-derived miR-9 effectively reduced SOCS5 expression and activated JAK-STAT pathway, which promoted endothelial cell migration and tumour angiogenesis. This pro-angiogenetic role of miR-9 was proved in several cancers, including lung cancer, melanoma, pancreatic cancer, glioblastoma and colorectal cancer [12]. Qu et al. indicated that miR-9 overexpression promoted osteoblast differentiation and angiogenesis via the AMPK signaling pathway [24]. Another research found that miR-9 coupled neurogenesis and brain angiogenesis through the inhibition of Tlx and Onecut transcription factors regulating neuronal VEGF-A expression [25]. Although regulation of proliferation, epithelial-mesenchymal transition, invasion, and tumor angiogenesis attributed to miR-9 is studied in a variety of cancer types [26], the effect of miR-9 on angiogenesis in NPC have never been elucidated. Our data showed that exosomal miR-9 enhanced recipient HUVEC cell migration and tube formation. Additionally, we found that the plasma exosomal miR-9 level was positively correlated with overall survival in NPC patients, suggesting the clinical relevance and prognostic value of tumor-derived exosomes.

Midkine (MDK) is a heparin-binding growth factor prominently expressed in embryonic tissues but down-regulated to negligible levels in healthy tissues of adults, which is considered to be a “pan-cancer” biomarker [27–29]. Overexpression of MDK were observed in many cancers [29–31], including lung cancer, ovarian carcinoma and thyroid carcinoma. However, the status and function of MDK have never been documented in NPC tumorigenesis. In this study, we found that MDK was a new target gene of miR-9 in endothelial cells. Previous studies showed that MDK activated both the Akt and ERK1/2 pathways, upregulated the expression of several cell-cycle-related proteins and promoted gastric cancer cell survival and growth [32]. Another study found that MDK activated AKT and ERK pathways and induced the Adriamycin resistance in gastric cancer cell [33]. Our results indicated that the inhibitory effect of exosomal miR-9 on endothelial cell was mediated by targeting MDK and regulating PDK1/AKT pathway. For the first time, we illustrated the link between MDK and PDK1/AKT pathway in tumor angiogenesis. More importantly, we found that MDK was overexpressed and positively correlated with microvessel density in NPC, and high MDK expression was positively associated with poor prognosis in NPC, suggesting the valuable potential of MDK as a prognostic biomarker.

This study has several limitations. Tumor angiogenesis is known to be modulated by multiple kinds of factors, including cytokines, chemokines, and growth factors derived from tumor microenvironment [34]. Exosomes carry various molecules, including proteins, mRNAs and miRNA. Thus, further studies are needed to understand how soluble factors and/or exosomal contents affect miR-9 in the tumor microenvironment in NPC. There is another issue that needs to be addressed. The role of MDK in NPC tumorigenesis is required to be further elucidated in future. Most importantly, the clinical relevance of exosomal miR-9 and MDK expression in NPC patients awaited further validation on larger sizes of samples.

Taken together, this study demonstrated that exosomal miR-9 inhibited angiogenesis by targeting MDK and through PDK/AKT pathway in NPC. Given the molecular and biological complexity of angiogenesis, a better understanding of how cancer-derived exosomes participate in this process represents an important challenge, which can open new paths for the development of novel and effective anti-angiogenic drugs.

## Conclusions

Taken together, our data identify exosomal miR-9 derived from NPC cells inhibits angiogenesis by targeting MDK and regulating PDK/AKT pathway in nasopharyngeal carcinoma. The findings of this study suggest that miR-9 and MDK may be valuable as novel targets for the treatment of human nasopharyngeal carcinoma.

## Additional files

Additional file 1: **Table S1.** Patient and disease characteristics. (DOC 48 kb)

Additional file 2: **Figure S1.** 5-8F and CNE1 cell lines were transfected with LV-miR-9. (**A**) Representative images observed by visible light or fluorescence microscope of 5-8F cells after stable transfection with Lv-miR-9 and Lv-control, original magnification: × 100). (**B**) Representative images observed by visible light or fluorescence microscope of CNE1 cells after stable transfection with Lv-miR-9 and Lv-control, original emagnification: × 100). (**C**) miR-9 expression was significantly upregulated after Lv-miR-9 transfection in 5-8F and CNE1 cells. (TIF 1757 kb)

Additional file 3: **Figure S2.** miR-9-overexpressing exosome treatment significantly inhibited cell migration and tube formation of HUVEC compared with control. (**A**) miR-9 expression was significantly upregulated after miR-9 mimic transfection in exosome derived from 5-8F cells. (**B**) HUVEC cells were treated with the respective exosomes, with or without miR-9 overexpression, and cell migration was measured using Transwell migration assay. (**C**) Following miR-9-overexpressing exosomes treatment, tubule formation of HUVECs was examined by in vitro tube formation assay and quantified for tubule length. **, *P* < 0.01. (TIF 411 kb)

Additional file 4: **Figure S3.** MDK was negative in exosomes derived from 5-8F/con, 5-8F/miR-9, CNE1/con and CNE1/miR-9 cells. The protein level of MDK in exosomes derived from 5-8F/con, 5-8F/miR-9, CNE1/con and CNE1/miR-9 cells respectively measured by immunoblot. The intensity of each band was normalized by GAPDH. (TIF 1654 kb)

Additional file 5: **Figure S4.** MDK expression was significantly downregulated after siMDK transfection in HUVEC cells. (**A**) The mRNA level of MDK in HUVEC cells after siMDK transfection. (**B**) MDK protein expression levels in HUVEC measured by immunoblot after siMDK transfection. The intensity of each band was normalized by GAPDH. (TIF 220 kb)

Additional file 6: **Figure S5.** Ectopic expression of miR-9 significantly reversed MDK-induced promotion of cell migration and tube formation. (**A**) HUVEC cells were infected with LV-MDK for 72 h and followed by treatement with miR-9-ovexpressing exosome. The mRNA levels of MDK in HUVEC were examined using qRT-PCR. (**B**) The protein levels of MDK were measured by western blot. The intensity of each band was normalized by GAPDH. (**C**) Cell migration was measured and quantified by Transwell migration assay. (**D**) Tubule formation of HUVECs was examined by in vitro tube formation assay and quantified for tubule length. **, *P* < 0.01. (TIF 575 kb)

Additional file 7: **Figure S6.** AR-12 treatment significantly reversed MDK-induced promotion of cell migration and tube formation. (**A**) HUVEC cells were infected with LV-MDK for 72 h and followed by treatement with AR-12. Cell migration was measured and quantified by Transwell migration assay. (**B**) Tubule formation of HUVECs was examined by in vitro tube formation assay and quantified for tubule length. **, *P* < 0.01. (TIF 2679 kb)

## Abbreviations

3′-UTR: 3′: Untranslated region
HUVEC: Human umbilical vein endothelial cell
NPC: Nasopharyngeal carcinoma
