# Supplementary material for: Exosomal miR-9 inhibits angiogenesis by targeting MDK and regulating PDK/AKT pathway in nasopharyngeal carcinoma
Source: J Exp Clin Cancer Res. 2018 Jul 13;37:147. doi: 10.1186/s13046-018-0814-3 (PMC6044044; doi:10.1186/s13046-018-0814-3)
Supplement: Supplementary file 1 — Table S1. Patient and disease characteristics. (DOC 48 kb) [file 13046_2018_814_MOESM1_ESM.doc]

Additional file 1: Table S1. Patient and disease characteristics.

| Characteristics |  |
| --- | --- |
| Age, years | 46.04±12.07 |
| Gender |  |
| Male, n (%) | 83 (75.5%) |
| Female, n (%) | 27 (24.5%) |
| T stage |  |
| T1 | 27 (24.5%) |
| T2 | 24 (21.8%) |
| T3 | 28 (25.5%) |
| T4 | 31 (28.2%) |
| N stage |  |
| N0 | 11 (10.0%) |
| N1 | 35 (31.8%) |
| N2 | 50 (45.5%) |
| N3 | 14 (12.7%) |
| M stage |  |
| M0 | 106 (96.4%) |
| M1 | 4 (3.6%) |
| UICC stage |  |
| I | 7 (6.4%) |
| II | 19 (17.3%) |
| III | 39 (35.5%) |
| IV | 45 (40.9%) |
| Death |  |
| No | 79 (71.8%) |
| Yes | 31 (28.2%) |
