# Supplementary figures and images for: Exosomal miR-9 inhibits angiogenesis by targeting MDK and regulating PDK/AKT pathway in nasopharyngeal carcinoma
Source: J Exp Clin Cancer Res. 2018 Jul 13;37:147. doi: 10.1186/s13046-018-0814-3 (PMC6044044; doi:10.1186/s13046-018-0814-3)

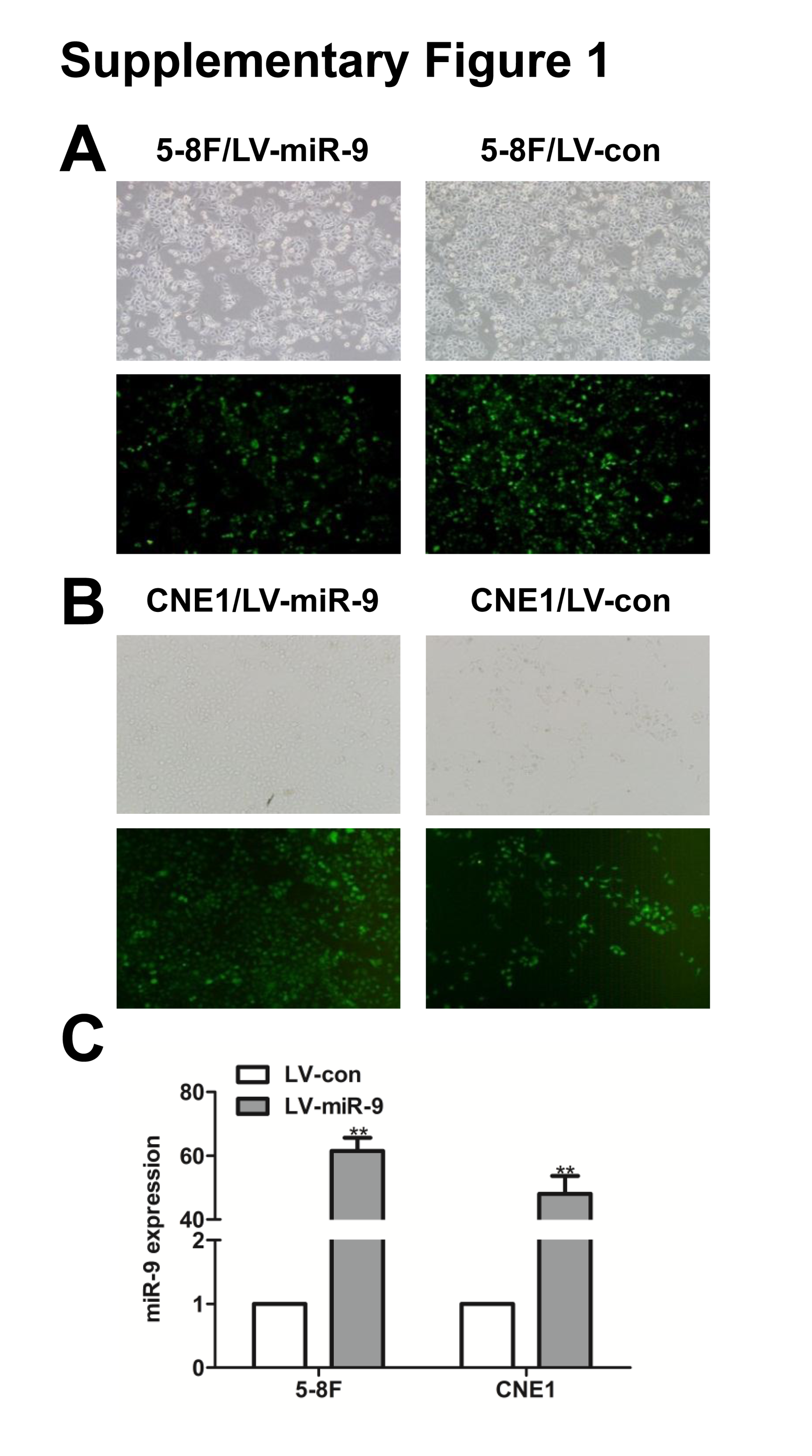

Supplement: Supplementary file 2 — Figure S1. 5-8F and CNE1 cell lines were transfected with LV-miR-9. (A) Representative images observed by visible light or fluorescence microscope of 5-8F cells after stable transfection with Lv-miR-9 and Lv-control, original magnification: × 100). (B) Representative images observed by visible light or fluorescence microscope of CNE1 cells after stable transfection with Lv-miR-9 and Lv-control, original emagnification: × 100). (C) miR-9 expression was significantly upregulated after Lv-miR-9 transfection in 5-8F and CNE1 cells. (TIF 1757 kb) [file 13046_2018_814_MOESM2_ESM.tif]

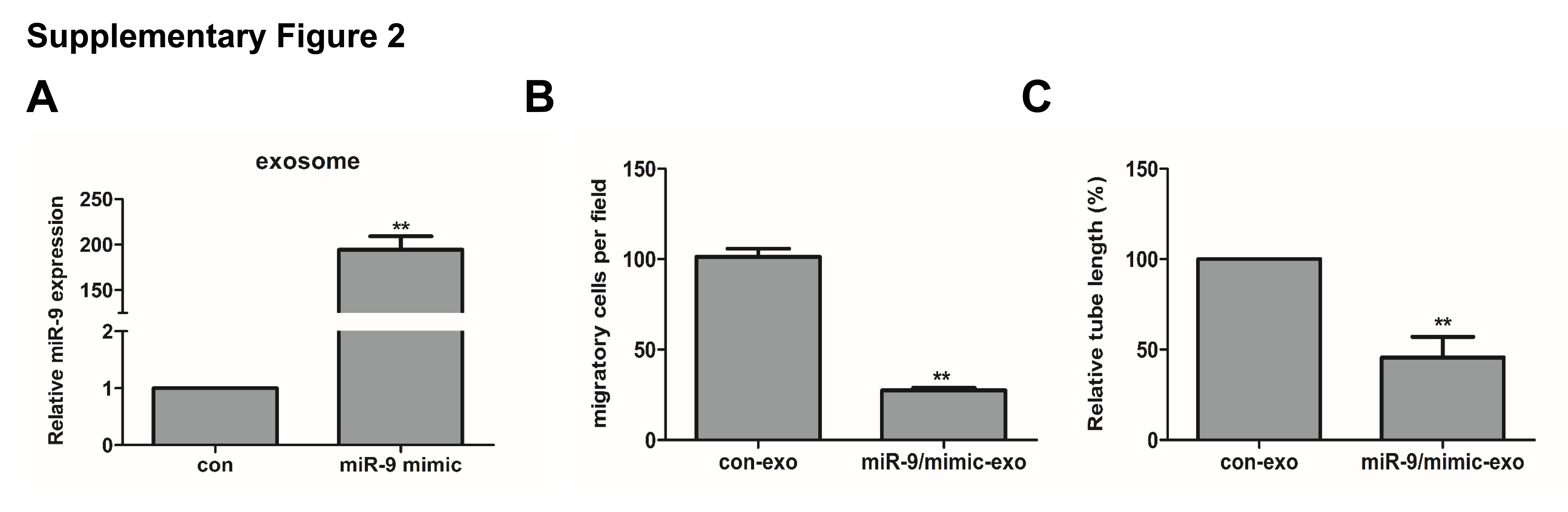

Supplement: Supplementary file 3 — Figure S2. miR-9-overexpressing exosome treatment significantly inhibited cell migration and tube formation of HUVEC compared with control. (A) miR-9 expression was significantly upregulated after miR-9 mimic transfection in exosome derived from 5-8F cells. (B) HUVEC cells were treated with the respective exosomes, with or without miR-9 overexpression, and cell migration was measured using Transwell migration assay. (C) Following miR-9-overexpressing exosomes treatment, tubule formation of HUVECs was examined by in vitro tube formation assay and quantified for tubule length. **, P < 0.01. (TIF 411 kb) [file 13046_2018_814_MOESM3_ESM.tif]

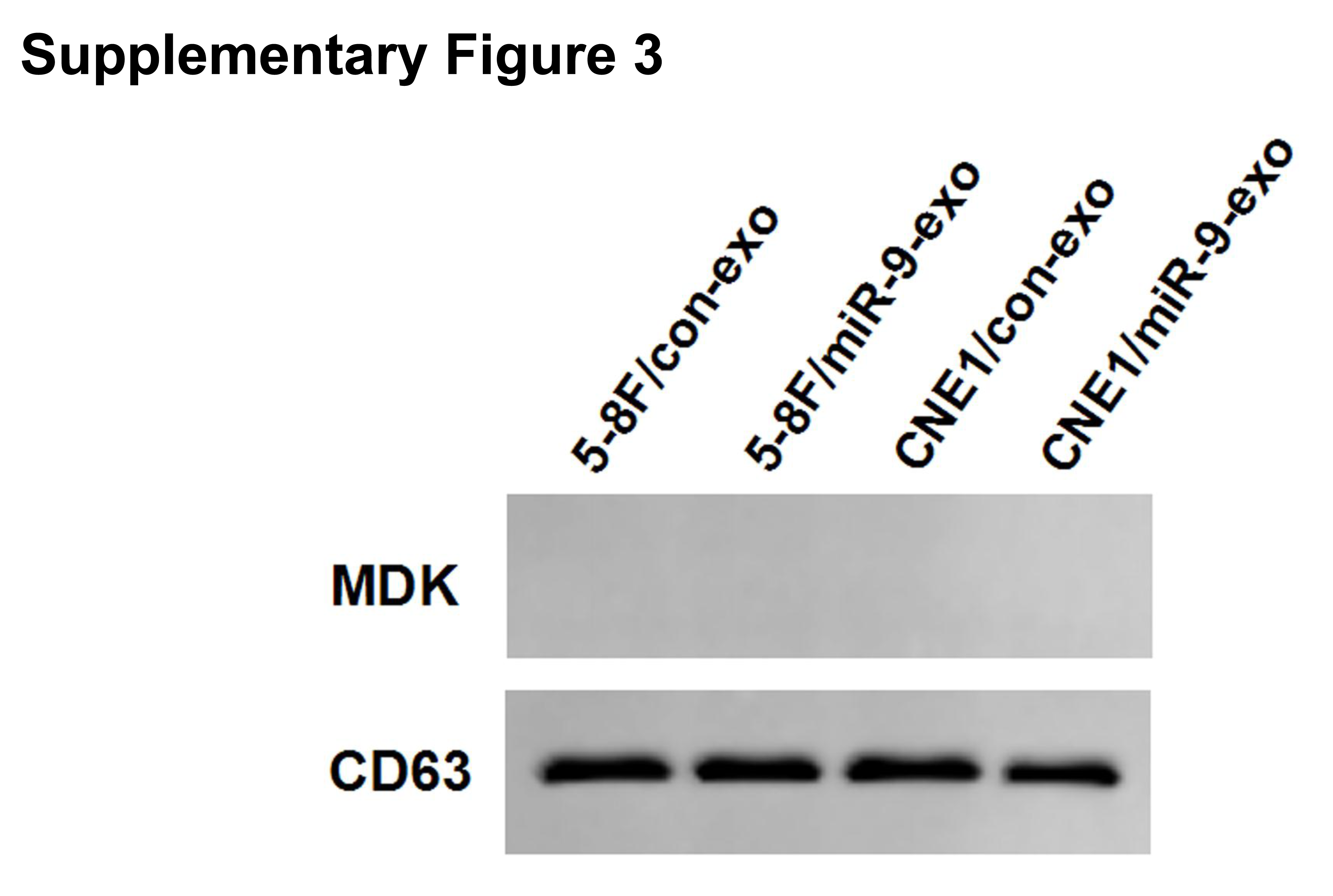

Supplement: Supplementary file 4 — Figure S3. MDK was negative in exosomes derived from 5-8F/con, 5-8F/miR-9, CNE1/con and CNE1/miR-9 cells. The protein level of MDK in exosomes derived from 5-8F/con, 5-8F/miR-9, CNE1/con and CNE1/miR-9 cells respectively measured by immunoblot. The intensity of each band was normalized by GAPDH. (TIF 1654 kb) [file 13046_2018_814_MOESM4_ESM.tif]

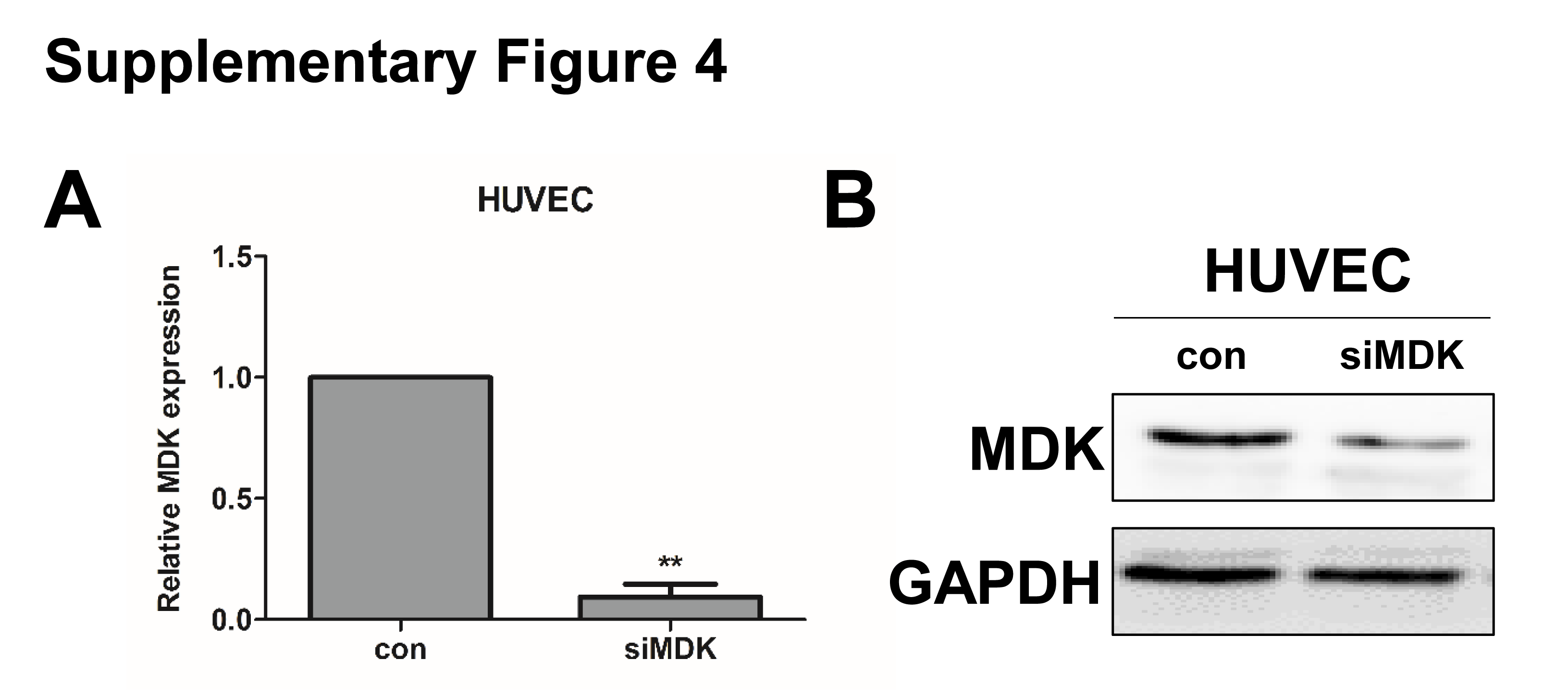

Supplement: Supplementary file 5 — Figure S4. MDK expression was significantly downregulated after siMDK transfection in HUVEC cells. (A) The mRNA level of MDK in HUVEC cells after siMDK transfection. (B) MDK protein expression levels in HUVEC measured by immunoblot after siMDK transfection. The intensity of each band was normalized by GAPDH. (TIF 220 kb) [file 13046_2018_814_MOESM5_ESM.tif]

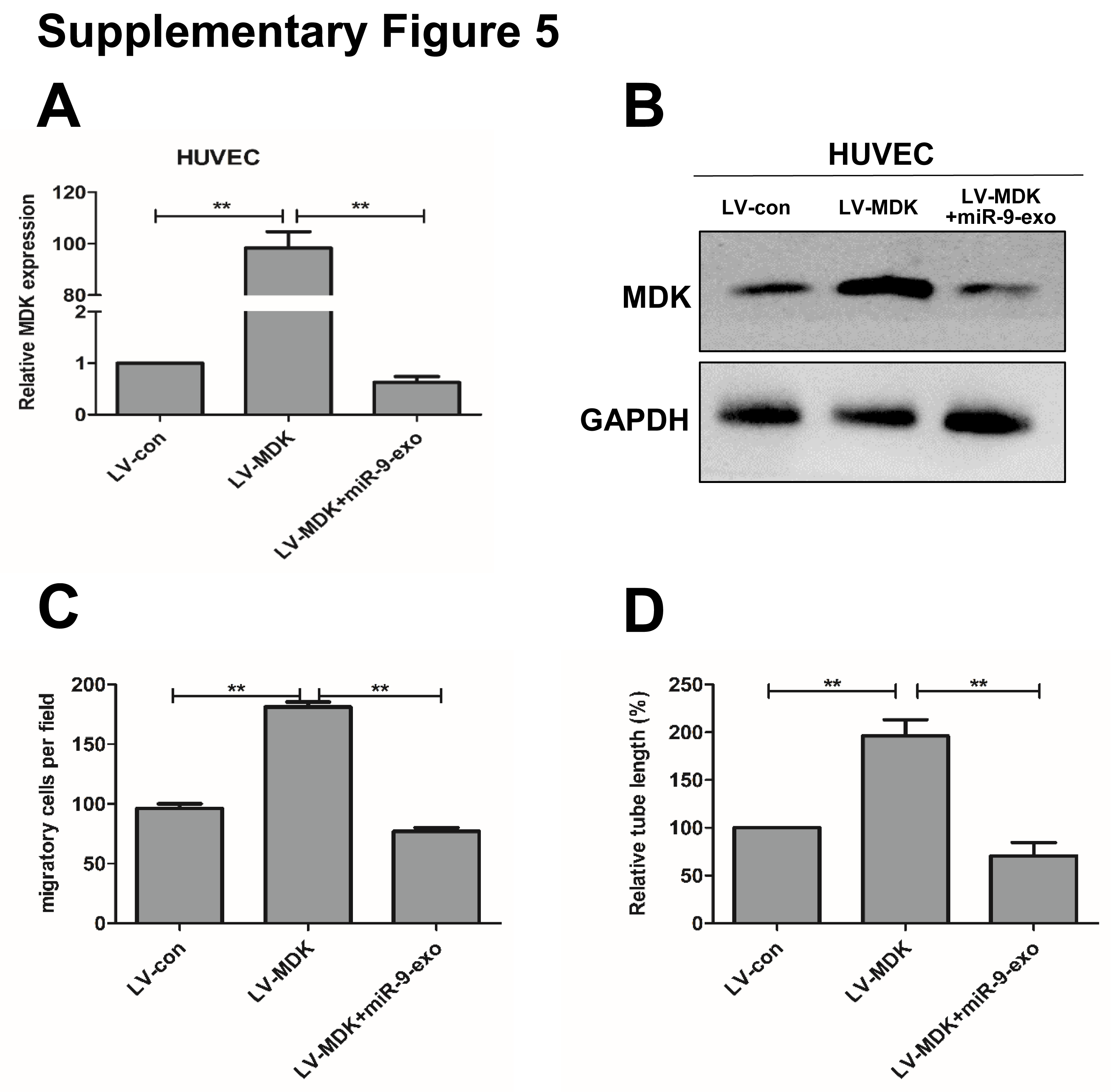

Supplement: Supplementary file 6 — Figure S5. Ectopic expression of miR-9 significantly reversed MDK-induced promotion of cell migration and tube formation. (A) HUVEC cells were infected with LV-MDK for 72 h and followed by treatement with miR-9-ovexpressing exosome. The mRNA levels of MDK in HUVEC were examined using qRT-PCR. (B) The protein levels of MDK were measured by western blot. The intensity of each band was normalized by GAPDH. (C) Cell migration was measured and quantified by Transwell migration assay. (D) Tubule formation of HUVECs was examined by in vitro tube formation assay and quantified for tubule length. **, P < 0.01. (TIF 575 kb) [file 13046_2018_814_MOESM6_ESM.tif]

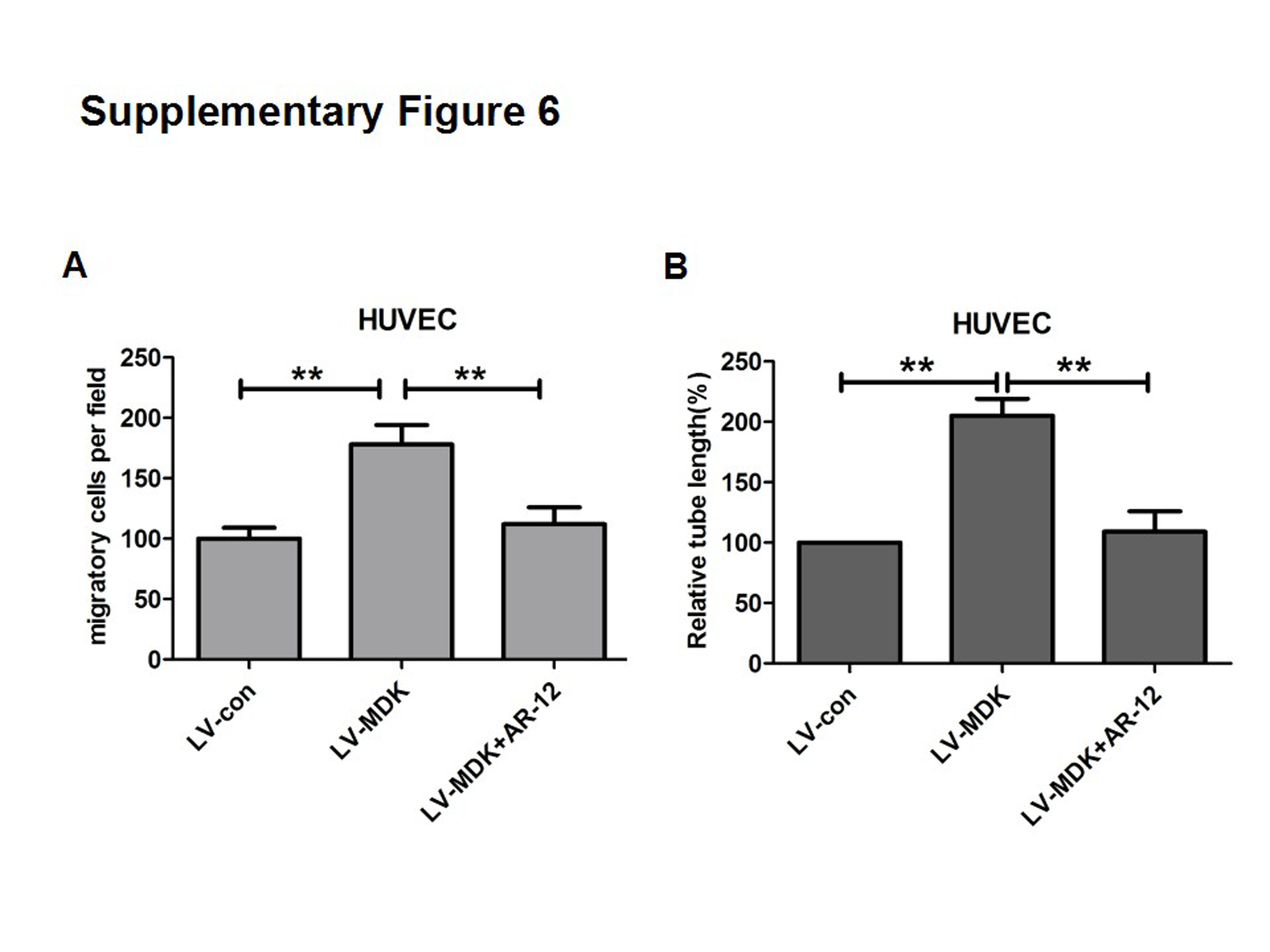

Supplement: Supplementary file 7 — Figure S6. AR-12 treatment significantly reversed MDK-induced promotion of cell migration and tube formation. (A) HUVEC cells were infected with LV-MDK for 72 h and followed by treatement with AR-12. Cell migration was measured and quantified by Transwell migration assay. (B) Tubule formation of HUVECs was examined by in vitro tube formation assay and quantified for tubule length. **, P < 0.01. (TIF 2679 kb) [file 13046_2018_814_MOESM7_ESM.tif]
